# Serum-free media for mesenchymal stem cells expansion on microcarriers

**DOI:** 10.1186/1753-6561-9-S9-P70

**Published:** 2015-12-14

**Authors:** Céline Martin, Alexandre Piccini, Isabelle Chevalot, Eric Olmos, Emmanuel Guedon, Annie Marc

**Affiliations:** 1 Laboratoire Réaction et Génie des Procédés, UMR 7274, CNRS-Université de Lorraine, 2 avenue Forêt de Haye, TSA 40602, 54518 Vandœuvre-lès-Nancy Cedex, France

## Background

Expansion of mesenchymal stromal cells (MSC) is one of the key steps for their use in tissue engineering or cell therapies. To increase cells expansion yields, two milestones have to be achieved that will allow a wider MSC therapeutic use, namely an optimal serum-free medium and a process intensification via 3D suspension culture on microcarriers [1]. Indeed, one of the major obstacles to obtain a reliable manufacturing process is that most of MSC cultivation methods still rely on media being supplemented by a significant volume of fetal calf serum. While efforts have been made to develop serum-free media (SFM) for MSC expansion, they were systematically designed for planar plastic cultivation systems. The aim of this study was to compare usual serum alternatives on 2D and 3D cultures. However, these alternatives can be ill-defined, either when the medium formulation is proprietary or when it has been humanized with blood derivatives. Thus several supplements were also investigated to design a more defined serum-free formulation that could be used to expand stem cells on microcarriers.

## Materials and methods

We performed batch cultures in 2D (static culture plates) and 3D (microcarriers) of human MSCs. Cells were first isolated from bone marrow by adhesion to plastic and validated a posteriori as MSC following ISCT recommendations. Negative control for cell expansion consisted in α-MEM + FGF2, ß-mercaptoethanol, and Glutamax. FCS was added for the positive control at 10 % v/v. Commercial media were purchased from Gibco, Lonza and Biological Industry, the coating solution CellStart from Gibco, and peptones from Kerry. SFM01 contains α-MEM/F12 supplemented with Glutamax, Hepes, r-insulin, h-transferrin, putrescin, progesterone, BSA, FGF2,TGF- α1, ß-mercaptoethanol, hydrocortisone, Lipid Concentrate and fetuin. Whatever the medium used, 1 % (v/v) antibiotics was added On the basis of cell growth monitoring, 2D cultures first allowed us to screen well-performing SF commercial media and more than a dozen of usual cell culture supplements using an automated microscopic follow-up with the Cell screen device (Roche). In a second time, 3D cultures on microcarriers at small-scale were performed. Cell adherence was first assessed on GE Healthcare® and Solohill® microcarriers, and only the best conditions were brought to cell expansion in shake flasks allowing us to monitor glucose/lactate kinetics in parallel with the cell growth. ). Density on microcarriers was determined by Guava cytometer. Two wells were sacrificed daily on 24-wells plates and a 0.6 mL sample was taken out of each shaking flask for triplicate measurements. Design of experiment was obtained using the Modde 7 software (D-optimal design screening with PLS model). Smoothing used the Savitzky-Golay algorithm.

## Results

**Figure 1:**
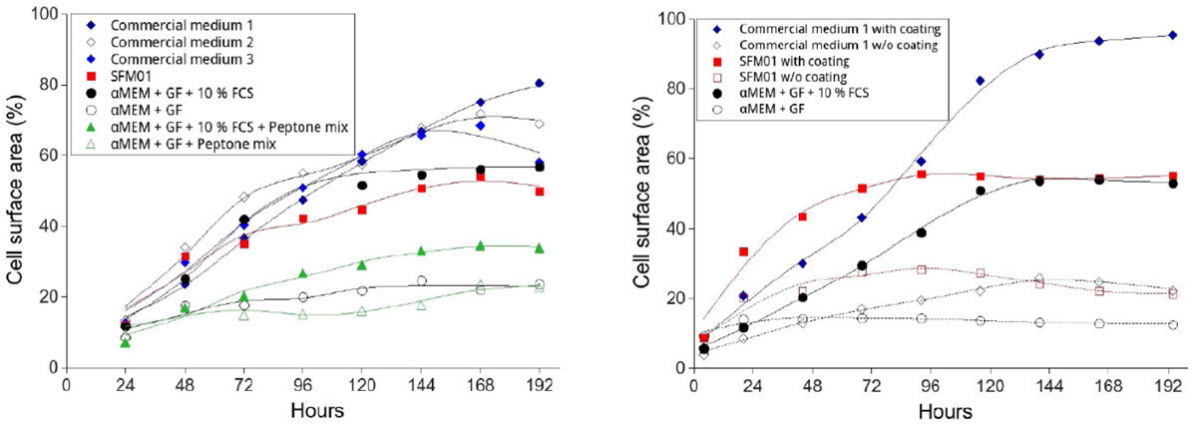
**Effect usual serum alternatives on the growth of hMSC cultivated in serum-free and commercial media**.

A first serum-free medium (SFM01) based on α-MEM/F12 and adapted from [2] was made by adding several components (see methods) in order to favor MSC adhesion and/or growth and was compared with commercial media, α-MEM/F12 supplemented with FCS (10%), growth factors and plant peptones in MSC 2D (figure 1) and 3D cultures.

Evidence indicated that adherence factors can be directly provided by supplementing the media or using adequate pre-coated microcarriers.

In order to improve SFM01 (i.e. SFM02 and SFM03), design of experiment was performed by testing 74 conditions including combination of 11 supplements added to the basal medium. In fact, most SF media still contain animal/human derivative. Therefore, several of them here we tested independently and in comparison with plant and yeast derivatives. On the basis of MSC growth measurement, the Table 1 summarized the major effect of components added in SFM 02 and SFM03.

**Table 1:** Effect of added components in serum-free media on MSC growth.

MSC growth	+	-
**Major effect**	Gelatin	B-27^®^

**Coupled effect**	Estradiol + IGF1 Estradiol + Lectin Laminin + Glutamax Laminin + Gelatin SyntheChol™+ B-27^®^ Fibronectin + B-27^®^	Fibronectin + Estradiol Serotonin + Estradiol SyntheChol™ + Gelatin

## Conclusions

Even though commercial media performed well in head-to-head comparisons, a defined serum-free medium remains necessary for a better understanding of MSC physiology, an increase in process robustness. However commercial SF media required a full renewing of the medium volume regularly and the necessity to use proprietary expensive cell coating reagents or using adequate pre-coated microcarriers (Cytodex 3, GE Healthcare). Indeed, adhesion factors are key components, particularly in stirred culture scale. Ideally, they should be provided directly in the medium as coating solutions in a serum-free process. In addition, identification of the most appropriates component and their compatibility with the adhesion substrate is still a major issue.

## Acknowledgements

This work was financially supported by the by the french ministry for higher education and research.
